# Stakeholders’ Experiences and Preferences Regarding Comprehensive Sexuality Education in Zambia: Implications for Determining Programme Priorities and Strategies

**DOI:** 10.3390/ijerph21081043

**Published:** 2024-08-08

**Authors:** Bright Mukanga, Siyabonga Blessing Dlamini, Myra Taylor

**Affiliations:** 1 Discipline of Public Health Medicine, School of Nursing and Public Health, University of KwaZulu-Natal, Durban 4001, South Africa; dlaminis24@ukzn.ac.za (S.B.D.); taylor@ukzn.ac.za (M.T.); 2 Public Health Department, Michael Chilufya Sata School of Medicine, Copperbelt University, Ndola P.O. BOX 71769, Zambia; 3 Cancer & Infectious Diseases Epidemiology Research Unit, College of Health Sciences, University of KwaZulu-Natal, Durban 4001, South Africa

**Keywords:** reproductive and sexual health, comprehensive sexuality education, adolescents, stakeholders

## Abstract

Background: Understanding and exploring stakeholders’ perceptions and preferences regarding comprehensive sexuality education (CSE) is critical in enhancing programme acceptability. We conducted this qualitative study to explore stakeholders’ experiences and preferences of CSE in Kitwe district, Zambia. Methods: We employed a qualitative descriptive design within the interpretive paradigm at nine purposefully selected secondary schools. Data were collected through classroom observation, in-depth interviews, and focus group discussions. In depth interviews were undertaken among 21 pupils, 17 teachers, 4 policymakers, 4 parents, and 4 religious leaders. Two focus group discussions were conducted among 14 pupils with each group having seven pupils. Data were analysed using inductive thematic analysis. Interviews continued until data saturation. Results: Our analysis yielded themes on stakeholder experiences of CSE which included: a lack of pupil-centred pedagogy; a lack of stakeholder engagement; a lack of CSE competency and skills; holding back culturally sensitive topics; a lack of guidance from the comprehensive sexuality curriculum; and a lack of CSE prioritisation in schools. Themes on preferences included: the formation of community advisory boards; the need to enhance teachers’ professional competencies, linking CSE with community SRH services, pupils SRH needs assessment. Conclusions: A community participatory approach to the design and implementation of a CSE programme is critical in enhancing programme acceptability in schools. While understanding the experiences and preferences of pupils regarding CSE can help determine programme content and priority, improving teachers’ competency and skills through appropriate CSE training can help to reconcile teachers’ personal beliefs and the content of CSE.

## 1. Introduction

Comprehensive sexuality education (CSE) can enhance adolescents’ positive sexual and reproductive health (SRH) outcomes [1]. This is particularly important in low- and middle-income countries (LMICs) where adolescents continue to face SRH challenges such as gender-based violence, stigma and discrimination, and insufficient implementation of CSE, which increase adolescents’ vulnerability to the risk of acquiring human immunodeficiency virus (HIV) [2]. Despite long decades of efforts to reduce the HIV burden, the HIV prevalence has continued to increase among adolescent girls and other vulnerable minority groups [3]. Estimates indicate that about 480,000 (255,000–760,000) young people aged between 10 and 24 were newly infected with HIV in 2022, of whom 140,000 (35,000–250,000) were adolescents aged between 10 and 19 years [4]. To address RSH challenges and enhance access to accurate and timely sexual health information among adolescents, many countries have implemented CSE in schools [5,6].

CSE has been defined as an “age-appropriate, culturally relevant approach to teaching about sexuality and relationships by providing scientifically, accurate, realistic and non-judgement information” [7]. Several benefits have been associated with CSE implementation, which include delayed onset of sexual activity, increased use of contraception, and a reduction in risky sexual behaviours (RSB) such as unwanted pregnancies, multiple sexual partnerships, and transactional sexual relationships [2,8]. Kirby [9] noted that schools have the potential to reduce the risk of sexual behaviours because most adolescents spend much of their time in schools before they initiate sex [10]. Achora et al. [1] also stated that the school setting affords a better platform to communicate sexual health knowledge and skills without stigma and discrimination. However, implementation of CSE in many LMICs, including Zambia, has been patchy and has faced social and religious opposition from stakeholders such as teachers, parents, and religious leaders [3,11,12,13,14]. The opposition to CSE is premised on the fact that the content of CSE is not in congruence with local cultural norms. Invariably, teachers water down content which is perceived to be against religious and cultural beliefs [12]. Though the CSE framework in many LMICS is grounded on the rights-based approach to teaching sexuality education, issues on sexual diversity and identities are rarely discussed during CSE sessions, because they are considered culturally inappropriate [5,7,11,12,15].

Exploring stakeholders’ experiences and preferences during the design and implementation of a CSE programme is critical in determining programme priorities and strategies as well as bridging the CSE cultural divide [3,16]. Stakeholder engagement through community participation can elicit views and opinions from community members regarding the development and implementation of the CSE programme [16]. The formation of community advisory boards (CABs) forms a huge part of community participation and can include stakeholders such as religious leaders, parents, policymakers, traditional leaders, and civic leaders [16,17]. In addition to helping to develop a sense of programme ownership, a participatory approach to CSE implementation can enhance the diffusion of ideas and programme adoption in the community [18]. Importantly, stakeholder engagement should include the needs assessment and meaningful participation of pupils who are the primary beneficiaries of CSE, and pupils’ views and preferences should inform the content of the CSE programme [19]. That said, the effectiveness of a CSE programme hinges on the pupils’ ability to voice sexual reproductive health and rights (SRHR) needs and decisions to challenge exclusionary cultural norms and practices [20]. However, in many settings including Zambia, CSE has been counter-productive and reinforces gender stereotypes and discriminatory norms due to opposition to the notion of young people’s rights [21].

To address SRH challenges among adolescents, the revised Zambian CSE framework was rolled out in 2014 in all primary and secondary schools, in line with the UNESCO international guidelines [22,23]. This idea was motivated by the fact that the initial reproductive health education curriculum introduced in the 1990s was limited, as it did not cover essential SRH themes such as gender relations, sexual behaviours, contraception, values, attitudes, and self-efficacy [12]. The revised Zambian CSE framework is centred on delaying sexual debut, increasing sexual health knowledge, and building skills and positive attitudes among pupils from grades 5 to 12 [12]. Despite this policy gain, estimates show that in Zambia, 10% of female adolescents report first marriage by age 15 years, with 135 births per 1000 female adolescents reported in 2018 [24]. Teenage pregnancies among schoolgirls continue to be reported across the country, a total of 120,878 pregnancies among school-aged adolescent girls (of which 109,850 were from primary schools and 26,752 from secondary schools) were reported between 2011 and 2019 [25]. Additionally, Zulu et al. [12] reported that as many as 25% of married girls aged 15–19 years have an unmet need for family planning, with 30% of girls aged 15–19 years beginning childbearing.

In Zambia, the implementation of CSE in schools has faced social opposition among stakeholders such as teachers and parents. Zulu et al. [12] noted that teachers were implementing CSE with discretion, watering down content that conflicted with their religious and cultural beliefs. The political and social factors in Zambia largely shape which components of CSE are considered acceptable among stakeholders [15]. Furthermore, the CSE programme faced a backlash among civic organisations and policymakers, and there were suggestions for its removal from the Zambian education curriculum [15,26]. Against this backdrop, this study sought to explore the stakeholder perceptions and preferences regarding CSE implementation in Zambia. Findings from this study have implications for determining CSE programme priorities and strategies in Zambia and other LMICs.

## 2. Methods

### 2.1. Study Design

This was a qualitative descriptive design within the interpretive paradigm. This design was appropriate considering the subjective interpretation of sexuality and education concepts [27]. As such, the naturalistic inquiry allowed for inductive theory reconstruction in the participants’ natural settings about their different experiences of the CSE programme. An interpretive design is ideal for understanding how participants construct the concept of sexuality within their natural settings.

### 2.2. Study Setting

The study was conducted in nine purposefully selected public secondary schools in the Kitwe district of Zambia. The schools were selected to include different geographic areas (four urban, four peri-urban, and one rural). Kitwe district is in the Copperbelt province of Zambia with an HIV prevalence of 14.2% [12,28]. Although Kitwe district was among the first districts in Zambia to implement the CSE programme, the prevalence of adolescent pregnancy is 17% [29]. Adolescents also lack access to the much-needed comprehensive SRH services such as contraceptive methods to prevent pregnancy and condom negotiation skills [30].

### 2.3. Sampling and Target Population

For this study, we employed criterion purposeful sampling to select participants comprising grade 12 pupils (*n* = 35) aged between 16 and 20 years, teachers (*n* = 17), parents (*n* = 4), policymakers (*n* = 4), and religious leaders (*n* = 4). Criterion sampling is a type of purposeful sampling where information-rich participants who meet some predetermined criteria are selected [31]. As such, we selected grade 12 pupils who were members of sexual health clubs in various schools and parents and religious leaders who were members of the parent-teacher associations (PTA).

### 2.4. Inclusion and Exclusion Criteria

We included grade 12 pupils who had experience of learning CSE from grades 8 to 12 and were members of school based SRH clubs such as DREAMS, and grade 12 teachers who were teaching CSE. In addition, we included policymakers, religious leaders, and traditional leaders who were part of the implementation of CSE and were members of parent–teachers’ associations. Pupils from grades 8 to 11 were excluded from the study.

### 2.5. Data Collection

Data were collected through in-depth interviews (IDIs) (*n* = 50), focus group discussions (FGD) (*n* = 14) with seven participants in each group, and classroom observation. One focus group discussion was of the same sex (girls) whilst the other was mixed sex (boys and girls). The 50 IDIs comprised parents (*n* = 4), teachers (*n* = 17), policymakers (*n* = 4), pupils (*n* = 21), and religious leaders (*n* = 4). The IDI and FGD guides were developed by the first author with input from the other authors. We conducted FGDs to leverage the synergistic effects of group discussions to further solicit participants’ experiences of CSE. Data collection lasted four months, from January to April 2023. We engaged research assistants trained in qualitative research who were familiar with the local language (Bemba) to collect the data. The results from both IDIs and FGDs were discussed among all authors after which the FGD and IDI guides were further revised. The broad research question which guided the methodological assumption was centred on exploring the experiences and preferences of CSE implementation among stakeholders. Through IDIs and FGDs, we probed aspects of CSE cultural appropriateness, stakeholder engagement and experiences, CSE curriculum, and the challenges of CSE implementation in schools and the CSE mechanisms of change. The IDIs and FGDs were held in a secluded place away from the administration blocks but within each of the school premises to ensure privacy and confidentiality. Data collection continued until the saturation point was achieved. The IDIs lasted between 40 and 50 min, and the FGDs lasted for 60 min. We conducted classroom observations at nine schools to triangulate findings from the IDIs and FGDs and to identify aspects of CSE that teachers emphasised and whether participatory methods were being used by teachers.

### 2.6. Data Analysis

The audio recordings were transcribed verbatim, after which all the data sets were reviewed repeatedly by all members of the research team to familiarise ourselves with the data. We further coded the interview scripts using NVIVO software (NVvivo.x64). The codes were then merged into categories, and themes on preferences and perceptions of CSE were developed through an iterative process. Overall, this inductive analysis was based on the six multi-step thematic coding, which comprised familiarising with the data, producing initial codes, compiling codes into initial themes, defining themes and sub-themes, and discussing and member-checking themes [32,33]. The observation data were coded thematically.

### 2.7. Ethical Considerations

Approval for the study was granted by the Tropical Diseases Research Centre in Ndola, Zambia (IRB registration number: 00,002,911 and FWA number: 00003729) and permission was granted by the National Health Research Authority (NHRA), Zambia (ref No: NHRA000001/01/06/2022), and the Biomedical Research Committee of the University of KwaZulu-Natal in Durban, South Africa, approved the study (approval number: BRE/00004141/2022). Written informed consent was obtained from all participants before data collection. We followed the standard procedures of informed consent, which included anonymity and confidentiality. Before the actual data collection, letters of support were obtained from the district education office and all the participating schools. For adolescents under the age of 18 years, we obtained assent from their parents and guardians to participate in the study. The participants were informed about the purpose, benefits, and possible harms of the study. We anonymised all data records and ensured privacy and confidentiality.

### 2.8. Rigour of the Study

We employed Guba and Lincoln’s criteria of ensuring trustworthiness in qualitative research (i.e., credibility, dependability, transferability, and confirmability) to maintain the rigour of this study [34]. To enhance credibility, we spent about 8 weeks in the field, engaging with the participants in their natural settings to understand their experiences regarding CSE. After we transcribed the recordings, the participants were asked to cross-check and validate the transcribed recordings. Several peer debriefings were held where all members of the research team were asked to review and assess the transcripts, methodology, and research findings. To enhance dependability, we established a dependability audit whereby an independent qualitative researcher was engaged to review the data collection process that we had undertaken. Several triangulation techniques which included the methodology and data sources such as IDIs, FGDs, and classroom observations were employed. Transferability was enhanced through thick descriptions of the study methods and through detailed drafts of the study protocol [35]. As such, we ensured that data collection continued until no further theoretical insights were observed in the emerging data. To enhance the confidence that the results would be corroborated by other researchers, we conducted a confirmability audit trail where we highlighted a clear coding schema and data were checked and rechecked throughout the whole research process.

## 3. Results

In total, we had sixty-four (64) subjects who participated in the IDIs and FGDs. This comprised 35 pupils (19 boys, 16 girls) with a mean age (SD) of 17.5 (0.95) and 17 teachers (11 females, 6 males) with a mean age (SD) of 38.8 (7.3) across nine schools, four parents (two females, two males), four policymakers (two females, two males), and four religious leaders (two females, two males). (see Table 1).

### 3.1. Themes on Experiences and Preferences of CSE by Stakeholders

Ten themes emerged from the IDIs and FGDs on the experiences and preferences of CSE by stakeholders which included: lack of participatory and pupil centred pedagogy; lack of CSE competency and skills; holding back culturally sensitive topics, lack of guidance from the CSE curriculum; lack of CSE prioritisation in schools; and lack of stakeholder engagement .Themes on preferences regarding CSE included: linking CSE with community SRH services; enhancing teachers professional competencies; pupils SRH needs assessment and formation of community advisory boards (CABs) (see Table 2)

### 3.2. Themes on Stakeholders’ Experience of CSE

#### 3.2.1. Theme 1: Lack of Participatory and Pupil-Centred Pedagogy

The classroom observations revealed that CSE teaching lacked pupil-centred pedagogy with no participatory approaches included to enable pupils to reconcile their experiences and ideas with CSE. In many cases, pupils were passive onlookers during CSE lessons. Most teachers taught CSE for a few minutes citing challenges related to time. One pupil said this about the lack of participatory and learner-centred approaches to CSE delivery.

*“We normally just sit and listen, and lessons are not participatory. There is nothing such as role plays and drama. As such, it is difficult to ask questions about SRH challenges and cultural issues.”*

(pupil, 18 years old, Female, IDI)

When asked about participatory methods in CSE teaching, most teachers cited a lack of teaching time because CSE was taught in carrier subjects such as home economics, biology, and mathematics, and was not included on the school timetable.

*“As teachers, we are required to integrate CSE into other carrier subjects. So, it is very difficult for us to exhaust subjects in CSE due to lack of time. If CSE was a stand-alone subject and timetabled, it would have been easier for us to include participatory and student-centred approaches. Most of us just use the first five minutes of CSE to talk about CSE issues.”*

(teacher, 35 years old, Male, IDI)

#### 3.2.2. Theme 2: Lack of CSE Competency and Skills

The UNESCO international guidelines recommend teacher training in CSE because CSE comprises subtle topics which require pre-service training, ongoing professional development, and capacity building for teachers [23]. Though it was a policy requirement for all teachers to integrate CSE into carrier subjects, we found that many teachers were not adequately prepared to teach and integrate CSE. Partly, this was because most teachers had not received formal training in CSE integration, and in many schools, only guidance teachers had been trained in CSE. A male teacher had this to say regarding the lack of skills in teaching CSE.

*“As a geography teacher, how do I integrate sexuality issues in my subject? most of us lack the skills to integrate CSE because we have not been trained, only guidance teachers have been trained in most schools”*

(teacher, male, 36 years old, IDI).

Another female teacher questioned the integrity of the CSE integration policy in schools when most teachers had not received formal training in CSE. She cited that in most schools, CSE was just a concept on paper that had not been practically implemented.

*“This CSE thing is just on paper in most schools because most teachers are not practically implementing it. One wonders how the programme can be implemented without training teachers in CSE. Even though we have the CSE policy framework and teacher manuals, we do not have the skills to integrate CSE, say for instance, in subjects like mathematics and geography’’.*

(teacher, female, 38 years old, IDI).

#### 3.2.3. Theme 3: Holding Back Culturally Sensitive Topics

The classroom observations and IDIs revealed that teachers were holding back the content of the CSE programme that was against their religious and cultural beliefs. This was due to teachers’ failure to reconcile professional experiences and their religious and cultural beliefs. In doing so, some teachers focussed much on abstinence-only messages as a way of preventing pregnancy and sexually transmitted infections (STIs).

*“Yes, teaching CSE is mandatory, but it is very difficult for me to teach pupils values that are against my religious and cultural beliefs, so when teaching, I usually emphasise those aspects of the CSE programme that are in line with biblical beliefs such as abstinence as means of preventing pregnancy and diseases’’*

(teacher, female, 40 years old, IDI).

#### 3.2.4. Theme 4: A Lack of Guidance from the CSE Curriculum

Although it was a policy requirement to integrate CSE into other career subjects such as home economics, science, and biology, teachers expressed concern about the lack of guidance from the CSE curriculum on CSE integration. A review of the Zambian CSE framework showed a lack of guidance on CSE integration, particularly the time and frequency of teaching of CSE and the teachers’ role in CSE implementation.

*“I feel the impact of CSE is little because it is something that we normally teach in less than 10 min. Also, the programme is not on the timetable, and there is no curriculum to guide us on time and frequency of teaching”*

(teacher, female, 47 years old, IDI).

#### 3.2.5. Theme 5: Lack of CSE Prioritisation in Schools

In all nine schools, we observed that CSE was not placed on the school timetable and the frequency and duration of teaching were conducted at the discretion of each teacher. Teachers attributed this to CSE not being a stand-alone subject and the programme not being examinable. Pupils also expressed concern that there was no consistency in teaching CSE among teachers, which they attributed to a lack of prioritisation of the programme.

*They (teachers) don’t teach CSE regularly, and when they teach, it’s only for a few minutes. I feel the programme is not a priority and most teachers do not take it seriously as it is not examinable. You also find that there is no timetable for a teacher to follow.*

(pupil, male, 18 years old, IDI).

#### 3.2.6. Theme 6: Lack of Stakeholder Engagement

Findings from the IDIs revealed no stakeholder engagement during the development and implementation of CSE in schools. Some religious leaders and parents claimed that the programme had faced a backlash from community members due to the non-engagement of community leaders, such as parents and religious leaders, during the development and implementation of CSE. One religious leader said this.

*“If we had been engaged when the programme was being developed and implemented, issues of cultural inappropriateness would have been addressed. Now you will find that even the parents of the pupils were not engaged during the implementation of the programme. How do you expect the community to react in such a case”*

(religious leader, male, 47 years old, IDI)

### 3.3. Themes on Preferences of CSE by Stakeholders

#### 3.3.1. Theme 7: Formation of Community Advisory Boards (CABs)

Studies have stressed the importance of community advisory boards which comprise parents, traditional leaders, and religious leaders [36]. In the current findings, stakeholders such as parents opined that the formation of community advisory boards would represent diverse views on the programme and could guide the cultural adaptation of the programme.

*The government need to engage the communities through representatives such as religious leaders, traditional leaders and parents when deciding on the content of the CSE programme. This can help determine CSE content appropriateness in line with our social norms. But as it is now, the programme is a pure Western agenda.*

(Parent, Male, 44 years old IDI).

#### 3.3.2. Theme 8: Linking CSE with Community SRH Services

Policymakers expressed concern that the CSE programme was not linked to any sexual and reproductive health services in the district and that the CSE programme was not helping the learners much because even though CSE was taught in schools, there were no available SRH services that learners could access.

*“We need to have the CSE programme linked to reproductive and sexual health services in the community, which pupils should access. As it is now, most schools do not have existing SRH services, and it would be worthwhile to have these services provided by health facilities in the surrounding communities”*

(policymaker, female, 35 years old, IDI).

#### 3.3.3. Theme 9: Enhancing Teachers’ Professional Competencies

Teachers expressed concern about the lack of skills and training in CSE. They recognised the importance of skill-based teaching in CSE implementation in enhancing reflecting teaching. One teacher said this.

*“We hope many teachers will be trained in CSE, especially on how to integrate CSE. To realise positive outcomes among pupils, skill-based teaching is essential. Training in CSE would also help us to reconcile our personal and professional beliefs and the content of CSE. For now, I don’t see this programme yielding positive goals.”*

(teacher, male, 32 years old. IDI).

#### 3.3.4. Theme 10: Pupils’ SRH Needs Assessment

Assessing the SRH needs of pupils during the development of CSE is crucial in aiding programme priorities and appropriateness. Pupils cited concerns about the lack of their participation in the development of the CSE programme. One male pupil had this to say.

*“I feel the best way to address the SRH needs of pupils through CSE is to engage pupils through participatory approaches to understand pupils’ sexual needs, this can enhance pathways to challenge exclusionary and discriminatory cultural norms and practices.”*

(pupil, male, 28 years old, IDI).

## 4. Discussion

This study explored stakeholders’ perceptions and preferences regarding CSE implementation in Kitwe district, Zambia. Overall, stakeholders such as teachers, parents, and religious leaders were religiously and culturally conflicted about CSE. Strikingly, most pupils perceived the CSE programme to be relevant in enhancing SRH information which could not be easily accessed due to cultural barriers. Studies elsewhere have reported similar findings [5,7,11]. The plausible explanation could be that the socio-cultural context plays a huge role in defining and shaping how individuals conceptualise sexuality [37], by influencing ideas and providing rules of sexual conduct between men and women which shape knowledge, beliefs, and practises regarding sexuality [3]. Marielle et al. [37] noted that teachers’ perceptions of CSE highlight the interplay between the teachers’ beliefs and practises and how they recontextualise the CSE policy in the wider socio-economic context. This depicts how teachers could be potential barriers to successful CSE implementation by teaching CSE in ways not in line with the programme designer’s intentions [38].

Findings from this study corroborate research on the importance of assessing perceptions and preferences of sexuality education as influenced by social-cultural factors through community engagement [3,5,12,15,39,40]. Similarly, a Tanzanian study which explored young people’s perceptions of CSE programmes within their natural context highlights the importance of considering the contextual appropriateness of CSE guided by social and cultural realities as experienced by the people [41]. In this study, young people described school-based CSE as “not for use” and not in consonance with the realities of “the Swahili streets” and that the content of CSE was more aligned with Western culture and values [3,41]. Amon-Adjei [42] argues that amidst the cultural controversy surrounding CSE implementation in LIMCs, the voices and perceptions of the learners should be prioritised and guide the discussion on the form and content of sexuality education, because they are the main beneficiaries of the CSE programme.

To address the disconnect between the implementation of CSE and social-cultural values, Achen et al. [3] recommended co-creation to enhance effective CSE implementation. Co-creation entails collaborative actions and decision-making between policymakers and local stakeholders in setting the CSE agenda for implementation [43]. Co-creation is enhanced through a community-based participatory approach and can guide the development of practical, feasible, and appropriate strategies that address social needs through engagement with local people [44]. An example of a community-based participatory project is a Yathu-Yathu (“For us, by us”) project which employed a community engagement approach in the core designing of an SRH programme to identify needs and acceptable strategies regarding RSH information [45]. In congruence with this, in a study conducted in Uganda, the exclusion of lesbian, gay, bisexual, and transgender (LGBT) issues from the school-based sex education curriculum was based on the preferences and feedback from stakeholders such as teachers, pupils, and religious leaders [3]. However, this continues to be a challenge in the implementation of CSE because in many LMICs, enhancing a culturally appropriate CSE entails omitting issues around abortion and homosexuality.

Although the importance of establishing an appropriate CSE curriculum in informing and guiding the implementation of CSE has been highlighted [9], there is an empirical knowledge gap on a teacher’s role in CSE implementation [46,47,48]. A complete and well-designed CSE curriculum can guide teachers on the pedagogical process and the effectiveness of teaching CSE [11]. Kirby [9], affirms that a well-designed sexuality education curriculum is central to enhancing the dose, fidelity, and reach of CSE implementation. In Zambia, integration of CSE into other carrier subjects is a policy requirement among teachers, yet the CSE curriculum lacks clarity on the frequency and time of teaching [12]. Similar findings have been reported elsewhere [6,11]. As such, most teachers exercise discretion and interpretation of CSE in terms of when, what, and how to teach [12,37]. Lack of training in CSE among teachers compromises the quality of CSE implementation in schools because untrained teachers are likely to resist facilitative and adaptation methods [49]. Conversely, comprehensive teacher training in CSE could help teachers understand the dynamics of their class and address difficult relationships and cultural issues among pupils [50].

The formation of community advisory boards (CABs) is part of community participation where community members are engaged during the design and implementation of an intervention programme. This is critical to designing a culturally relevant and responsive CSE programme [16]. Community advisory boards are made up of different stakeholders such as religious leaders, traditional leaders, parents, policymakers, and civic leaders [16,17]. The roles of CABs during CSE implementation could be critical in making the CSE culturally congruent to meet the needs of pupils and teachers [17]. Formation of CABs is even more imperative when implementing programmes that involve sensitive issues such as sexual behaviour where community participation is integral for the diffusion of new sexual concepts among community members. In the context of CSE programme implementation, the formation of CABs could ensure that the opinions and voices of targeted community members are reflected in the programme to enhance its integrity [17]. Importantly, using the interventional mapping approach to assess the needs of pupils to inform the content of CSE has been recommended [19]. However, while in high-income countries CABs are usually established by the scientific community, in many LMICs, CABs are often established by lobbyists and activists with a specific agenda which poses several challenges [51]. These challenges include a lack of financial support, challenges on how to ensure proper training for community members [51], and a lack of consensus among CAB members on issues that require extensive discussion due to divergent opinions [17]. Zhao et al. [52], also noted challenges such as unstable and unbalanced power relationships between programme developers and local communities, and language barriers between researchers and community members. In particular, language barriers and mistrust tend to be huge challenges for foreign investigators and implementers [53]. Still, a community-based participatory approach which employs local knowledge to understand health problems could be essential in developing and implementing a context-based CSE programme [3].

Poor access to SRH services and information has been linked with high school attrition, early and unintended pregnancies (EUP), and other HIV risk behaviours [25]. A noteworthy finding of this study was that the programme was not linked to any SRH facilities in the communities. This has the potential to undermine the overall purpose of CSE in that pupils are not able to access SRH services such as contraception and safe abortion services. This is further compounded by the inherent stigma and social discrimination which exist in SRH facilities [25]. As such, linking CSE to SRH services in community health facilities should involve a collaborative approach with key stakeholders such as parents, and healthcare providers to address barriers to accessing SRH services [16]. Also, collaborative actions are essential for strengthening the links between CSE programmes and existing health structures and networks in the community [15]. These study findings should be understood in light of the following limitations: considering the sensitive topic of sexuality in many cultural settings in Zambia, the likelihood of socio-desirability bias cannot be ruled out where some participants might have provided inaccurate responses. We minimised this bias by triangulating the data sources such as participant observation and FGDs. Additionally, during the IDIs and FGDs, participants were encouraged not to answer questions they were not comfortable with. By ensuring data saturation, we enabled a thick data description which enhanced the credibility of our data.

## 5. Conclusions

Although most pupils had accepted the CSE programme, teachers, parents, and religious leaders were religiously and culturally conflicted about the implementation and integration of CSE. A community participatory approach to the planning, design and implementation of a CSE programme is critical in enhancing programme acceptability in schools. While understanding the experiences and preferences of pupils regarding CSE can help determine programme content and priority, improving teachers’ competency and skills through appropriate CSE training can help to reconcile teachers’ personal beliefs and the content of CSE.

## Figures and Tables

**Table 1:** Socio-demographic characteristics of participants (*n* = 64).

Study Sample	Characteristic	Number of Participants (*n* (%))
Pupils (*n* = 35) Mean age (SD)	Female Male	19 (54.3) 16 (45.7) 17.5 (0.95)
Teachers (*n* = 17) Mean age (SD)	Female Male	11 (64.7) 6 (35.3) 38.8 (7.3)
School (*n* = 9)	Peri-urban Urban Rural	4 (44.4) 4 (44.4) 1 (11.1)
Parents (*n* = 4)	Female Male	2 (50.0) 2 (50.0)
Policymakers (*n* = 4)	Female Male	2 (50.0) 2 (50.0)
Religious leaders (*n* = 4)	Female Male	2 (50.0) 2 (50.0)

**Table 2:** The main themes and sub-themes on stakeholders’ perceptions and preferences.

Main Themes	Sub-Themes
▪ Lack of participatory and pupil centred pedagogy	▪ No role play activities
	▪ No discussions held
	▪ No pupil engagement during lessons
▪ Holding back culturally sensitive topics	▪ Selecting CSE content to teach.
	▪ Teacher discretion in teaching CSE.
▪ Lack of CSE skills	▪ No training in CSE integration
	▪ Only guidance teachers trained in CSE.
▪ Lack of guidance from the CSE curriculum	▪ No guidance on the time of teaching CSE and methods of delivery.
	▪ No specification on the frequency of teaching CSE.
	▪ No specified role of a teacher in CSE in CSE implementation.
▪ Lack of CSE prioritisation	▪ CSE is not timetabled.
	▪ Integration of CSE in carrier subjects.
	▪ CSE not examinable.
▪ Lack stakeholder engagement	▪ No engagement of teachers, pupils, religious leaders and parents during the designing, planning, and implementation of CSE.
	▪ No formation of advisory boards during CSE implementation.
▪ Linking CSE to RSH services in the community	▪ Enhancing pupils’ access to RSH services in the community.
	▪ Addressing barriers to pupils’ access to RSH in health facilities
▪ The need for formation of community advisory boards (CABs)	▪ Developing and implementing CSE to fit the cultural context.
	▪ Engaging community representatives to participate in CSE development.
▪ Enhancing teachers’ professional competencies	▪ Skill-based CSE teaching.
	▪ Reflective CSE teaching
▪ Needs assessment of pupils SRH.	▪ Assessing the needs of the pupils to inform the programme content.

## Data Availability

The data used and analysed during the current study are available from the corresponding author upon reasonable request.
